# Improved Bearings-Only Multi-Target Tracking with GM-PHD Filtering

**DOI:** 10.3390/s16091469

**Published:** 2016-09-10

**Authors:** Qian Zhang, Taek Lyul Song

**Affiliations:** Department of Electronic Systems Engineering, Hanyang University, Ansan, Gyeonggi-do 15588, Korea; zq2013750509@hanyang.ac.kr

**Keywords:** nonlinear estimation, bearings-only measurement, multi-target tracking, Gaussian mixture measurements, passive sensor

## Abstract

In this paper, an improved nonlinear Gaussian mixture probability hypothesis density (GM-PHD) filter is proposed to address bearings-only measurements in multi-target tracking. The proposed method, called the Gaussian mixture measurements-probability hypothesis density (GMM-PHD) filter, not only approximates the posterior intensity using a Gaussian mixture, but also models the likelihood function with a Gaussian mixture instead of a single Gaussian distribution. Besides, the target birth model of the GMM-PHD filter is assumed to be partially uniform instead of a Gaussian mixture. Simulation results show that the proposed filter outperforms the GM-PHD filter embedded with the extended Kalman filter (EKF) and the unscented Kalman filter (UKF).

## 1. Introduction

Bearings-only multi-target tracking (MTT) [1,2,3] with clutter and missed detections is a challenging nonlinear problem. The filter for such a problem should not only deal with the nonlinearity of measurements, but also solve the measurement origin uncertainty of multiple targets. Bearings-only measurements [4] are often obtained by passive sensors, such as sonars, and contain a low level of information about detected targets, leading to the observability problem [5]. For bearings-only single-target tracking, the observable level can be evaluated by the Cramer–Rao lower bound (CRLB) [6,7]. However, it is difficult to evaluate the level of observability in a theoretical context for the bearings-only multi-target tracking with clutter and missed detections [8]. In order to overcome the non-observable problem, the sensor needs to outmaneuver the targets [5,9]. As the origin of a measurement is not known for target tracking in clutter, the tracking filter needs to have a proper track-to-measurement-association method [1] or something equivalent, such as symmetric measurement equations [10] and random finite sets [11].

For tracking targets using nonlinear measurements, the most popular method is the extended Kalman filter (EKF) [12]. It linearizes the nonlinear measurement function around the predicted target state under the assumption that the true target state is close enough to the predicted state. Then, the measurement update of the filter can be performed using the Kalman filter (KF) [12], which has a good performance for linear systems. The unscented Kalman filter (UKF) [13] applies the unscented transform instead of linearization to the nonlinear measurement function. The probability density functions (pdfs) are represented and propagated using several sigma points. The particle filter (PF) [14] represents the nonlinear pdfs by considering the amount of particles and can obtain better performances than the EKF and the UKF. However, particle filters have a heavy computational load. Apart from these nonlinear filters, many other filters exist for nonlinear systems, such as the cubature Kalman filter (CKF) [15], the shifted Rayleigh filter (SRF) [16] and the ensemble Kalman filter (EnKF) [17].

To handle the measurement origin uncertainty problem in MTT with clutters, many techniques have been developed. Many of these methods belong to the following categories: joint probability data association (JPDA) [18], multiple hypothesis tracking (MHT) [19,20] and random finite set (RFS) [11] based methods. The JPDA filters come from combing the probability data association (PDA) [1] filter with joint events and can only track fixed and known numbers of targets. To accommodate varied and unknown numbers of targets, the joint integrated PDA (JIPDA) [21] filter, which is able to estimate the probability of target existence, is proposed. To improve the tracking accuracy, a multi-scan multi-target tracking algorithm, the joint integrated track splitting (JITS), was developed in [22]. As the JIPDA and the JITS suffer from heavy computational load when tracking targets in mutual proximity, the linear multi-target IPDA (LMIPDA) and the linear multi-target ITS (LMITS) were proposed in [22,23], respectively. Besides, the iterative JIPDA (iJIPDA) [24] tracker is also a computationally-efficient algorithm for the MTT. The MHT filters attempt to maintain and evaluate a set of measurement hypotheses with high track scores. There are many versions of the MHT filter, and most of them can be grouped into two classes: the track-oriented class [25] and the measurement-oriented class [19]. The JPDA and MHT approaches are formulated via data association, which takes a large proportion of computational resources, while the RFS approach is an emerging paradigm and is established without data association. The RFS-based filters [26,27,28] treat multi-target states and measurements as the state finite set and the measurement finite set, respectively. The RFS-based filters try to estimate the target set based on the measurement set. In this way, the RFS-based filters are performed in a computational efficient way for the multi-target tracking.

Among RFS-based filters, the probability hypothesis density (PHD) [26] filter is a first moment approximation to the multi-target predicted and posterior densities. It propagates the target intensities without considering data associations between targets and measurements. As there is no close form to the PHD filter, a sequential Monte Carlo (SMC) or Gaussian mixture (GM) technique was implemented, which resulted in the SMC-PHD [29,30] filter or the GM-PHD [31] filter. For bearings-only MTT, it is difficult to apply the SMC-PHD filter because of the problem of extracting estimated target states. For this reason, the GM-PHD filter is considered in this paper. The GM-PHD filter associated with the EKF and the UKF are termed as GM-PHD-EKF and GM-PHD-UKF, respectively.

In the GM-PHD-EKF and the GM-PHD-UKF, the predicted intensity is modeled by a Gaussian mixture, and the likelihood function [12] is approximated using a single Gaussian distribution. The updated intensity of each filter is obtained after the predicted intensity is updated by the measurements. As bearings-only measurements suffer from severe nonlinearity, the likelihood function approximated by a single Gaussian is not accurate enough. Furthermore, the accuracy of the updated intensity cannot get enough improvements after the predicted intensity is updated by the inaccurate likelihood function of the measurements. In this paper, the Gaussian mixture measurements-PHD (GMM-PHD) filter is proposed to address bearings-only MTT with clutter and missed detections. To improve the tracking accuracy, the target intensities are approximated by Gaussian mixtures, and the likelihood function is also modeled by a Gaussian mixture in the GMM-PHD filter. In this way, the updated intensity of the GMM-PHD is much more accurate than those of the GM-PHD-EKF and the GM-PHD-UKF after the predicted intensity is updated by a more accurate likelihood function, which is modeled by Gaussian mixtures. The proposed filter is a nonlinear MTT algorithm that can address not only bearings-only measurements, but also other nonlinear measurements. For bearings-only MTT, the targets may appear at any place in the measurement space. In order to reduce the number of selected parameters and avoid the undesirable effect of poor parameter selection [8], the derivation of the GMM-PHD filter is processed with a partially-uniform target birth model [8,32], but not a Gaussian mixture birth model. In the simulation experiment, the performance of the GMM-PHD is compared with the GM-PHD-EKF and the GM-PHD-UKF in terms of the optimal subpattern assignment (OSPA) [33] distance, the OSPA localization, the OSPA cardinality and the average CPU time.

The rest of the paper is arranged as follows. The bearings-only MTT problem is presented in Section 2. The proposed GMM-PHD filter is derived in Section 3. A simulation study is given in Section 4. Finally, a conclusion is proposed in Section 5.

## 2. The Bearings-Only MTT Problem

In this paper, bearings-only MTT with one passive sensor on a maneuvering platform is studied. The targets obey the continuous white noise acceleration (CWNA) motion model [1,12]. The target motion model and the sensor measurement model in the 2D Cartesian coordinates case are considered in this section. For the target *t*, the target state $e_{k}^{t}$ with position $\left(x_{k}^{t},y_{k}^{t}\right)$ and velocity $\left({\dot{x}}_{k}^{t},{\dot{y}}_{k}^{t}\right)$ at time *k* is expressed as:

(1) $$e_{k}^{t}={\left[x_{k}^{t},y_{k}^{t},{\dot{x}}_{k}^{t},{\dot{y}}_{k}^{t}\right]}^{\prime }.$$

An RFS $X_{k}$ of target states can contain any number $N_{k}$ of targets at time *k* and is given by:

(2) $$X_{k}=\left\{e_{k,1}^{t},\cdots ,e_{k,N_{k}}^{t}\right\}.$$

Let ***χ*** denote the single target state space and F(χ) denote the set of all finite subsets of ***χ***, i.e., $e_{k,1}^{t},\ldots ,e_{k,N_{k}}^{t}\in \chi$ and $X_{k}\in F\left(\chi \right)$.

As the target *t* is assumed to follow the CWNA motion model, its dynamic model can be expressed as:

(3) $$e_{k}^{t}=\Phi e_{k-1}^{t}+\nu_{k-1},$$

where the state propagation matrix **Φ** is time-invariant,

(4) $$\Phi =\left[\begin{array}{cc} 1 & T\\ 0 & 1 \end{array}\right]⊗I_{2},$$

$\nu_{k-1}$ is a sequence of zero mean, white Gaussian process noises with covariance:

(5) $$Q_{k-1}=q\left[\begin{array}{cc} T^{4}/4 & T^{3}/2\\ T^{3}/2 & T^{2} \end{array}\right]⊗I_{2},$$

*T* is the sampling time, $I_{2}$ is the 2×2 identity matrix and *q* is the power spectral density (PSD) [1]. The sensor state $e_{k}^{s}$ is given as:

(6) $$e_{k}^{s}={\left[x_{k}^{s},y_{k}^{s},{\dot{x}}_{k}^{s},{\dot{y}}_{k}^{s}\right]}^{\prime }.$$

Each target *t* can be detected with the probability $P_{D,k}$ at time *k*. The sensor can generate a measurement when the target is detected. We use $\theta_{k}^{t}$ to denote the target measurement at time *k* for target *t*. The measurement from the radar is:

(7) $$w_{k}\overset{\Delta }{\;=}\theta_{k}^{t}=h\left(e_{k}^{t},e_{k}^{s}\right)+\varpi_{k},$$

where:

(8) $$h\left(e_{k}^{t},e_{k}^{s}\right)=\tan^{-1}\left(\frac{x_{k}^{t}-x_{k}^{s}}{y_{k}^{t}-y_{k}^{s}}\right),$$

$\varpi_{k}$ is zero mean, white Gaussian measurement noise with covariance:

(9) $$R_{k}=\sigma_{\theta }^{2},$$

that it is uncorrelated with $\nu_{k}$. Therefore, each target *t* can generate a measurement RFS $\Theta_{k}\left(e_{k}^{t}\right)$, which can be either $\left\{\theta_{k}^{t}\right\}$ (the target *t* is detectable) or ∅ (the target *t* is not detected). In addition, the sensor also produces false measurements, which form an RFS $K_{k}$ at each time *k*. Then, the MTT measurement RFS $W_{k}$ at time *k* can be expressed as:

(10) $$W_{k}=K_{k}\cup \left[\underset{e\in X_{k}}{⋃}\Theta_{k}\left(e\right)\right].$$

## 3. The Proposed GMM-PHD Filter

In RFS-based methods, the Bayesian recursion of multi-target posterior density is propagated in time as:

(11) $$p_{k|k-1}\;\left(X_{k}|W_{1:k-1}\;\right)\;=\;\int \;f_{k|k-1}\;\left(X_{k}|X\;\right)p_{k-1}\;\left(X|W_{1:k-1}\;\right)\;\mu_{s}\;\left(dX\;\right),$$

(12) $$p_{k}\left(X_{k}|W_{1:k}\right)=\frac{g_{k}\left(W_{k}|X_{k}\right)p_{k|k-1}\left(X_{k}|W_{1:k-1}\right)}{\int g_{k}\left(W_{k}|X\right)p_{k|k-1}\left(X|W_{1:k-1}\right)\mu_{s}\left(dX\right)},$$

where $f_{k|k-1}\left(X_{k}|X\right)$ and $g_{k}\left(W_{k}|X_{k}\right)$ denote the multi-target transition density and likelihood function, respectively. Here, $p_{k}\left(X_{k}|W_{1:k}\right)$ denotes the multi-target posterior, density and $\mu_{s}\;\left(dX\right)$ is an appropriate reference measure on F(χ) [31].

To reduce the computational intractability, a first moment approximation (the PHD filter) of the recursion is proposed in [26]. The general form of the PHD filter recursion (without target spawning) is given by:

(13) $$\begin{array}{c} v_{k|k-1}\;\left(e\right)\;=\;\int \;P_{S,k}\;\left(\eta \;\right)f_{k|k-1}\;\left(e|\eta \;\right)v_{k-1}\;\left(\eta \;\right)d\eta +\gamma_{k}\;\left(e\right), \end{array}$$

(14) $$\begin{array}{c} v_{k|k}\left(e\right)=\left[1-P_{D,k}\left(e\right)\right]v_{k|k-1}\left(e\right)+\sum_{w\in W_{k}}\frac{P_{D,k}\left(e\right)g_{k}\left(w|e\right)v_{k|k-1}\left(e\right)}{\kappa_{k}\left(w\right)+\int P_{D,k}\left(e\right)g_{k}\left(w|e\right)v_{k|k-1}\left(e\right)de}, \end{array}$$

where $v_{k|k-1}\left(e\right)$ and $v_{k|k}\left(e\right)$ denote the predicted intensity from time k-1 to *k* and the updated intensity, respectively, $P_{S,k}\left(\eta \right)$ is the survival probability, meaning the target still survives at time *k*, $f_{k|k-1}\left(e|\eta \right)$ represents the single target transition density from time k-1 to *k*, $\gamma_{k}\left(e\right)$ denotes the prior intensity of spontaneous target births at time *k*, $g_{k}\left(w|e\right)$ is the single target measurement likelihood function and $\kappa_{k}\left(w\right)$ is the clutter intensity.

### 3.1. Intensity Prediction

According to the Gaussian mixture assumption, the posterior intensity at time k-1 can be expressed as:

(15) $$\begin{array}{c} v_{k-1}\left(e\right)=\sum_{i=1}^{J_{k-1}}\omega_{k-1}^{\left(i\right)}N\left(e;{\hat{e}}_{k-1}^{\left(i\right)},J_{k-1}^{\left(i\right)}\right). \end{array}$$

Let θ,r,c,ands denote the bearing, range, course and speed in polar coordinates, respectively. The function $\Psi \left(e;\gamma_{k}\left(\theta ,r,c,s\right)\right)$ represents the transformation of the density $\gamma_{k}\left(\theta ,r,c,s\right)$ from polar coordinates to Cartesian coordinates. Then, the predicted intensity at time *k* is given by:

(16) $$\begin{array}{c} v_{k|k-1}\left(e\right)=v_{S,k|k-1}\left(e\right)+\Psi \left(e;\gamma_{k}\left(\theta ,r,c,s\right)\right), \end{array}$$

where:

(17) $$\begin{array}{c} v_{S,k|k-1}\left(e\right)=P_{S,k}\sum_{i=1}^{J_{k-1}}\omega_{k-1}^{\left(i\right)}N\left(e;\;{\hat{e}}_{k|k-1}^{\left(i\right)},J_{k|k-1}^{\left(i\right)}\right), \end{array}$$

(18) $$\begin{array}{c} \gamma_{k}\left(\theta ,r,c,s\right)\;=\omega_{k}^{b}U\;\left(\theta ;H_{\theta }\right)N\left(r;\bar{r},\sigma_{r}^{2}\right)N\left(c;\theta -\pi ,\sigma_{c}^{2}\right)N\left(s;\bar{s},\sigma_{s}^{2}\right). \end{array}$$

In Equation (17), the survived probability $P_{S,k}\left(e\right)$ is assumed to be independent of the target state and is given as $P_{S,k}$. In Equation (18), $\omega_{k}^{b}$ is the expected number of targets appearing at time *k*, $U\left(\theta ;H_{\theta }\right)$ is the uniform distribution in *θ* over the region $H_{\theta }$, $\bar{r}$ is the prior mean of the range and $\sigma_{r}^{2}$ is its prior variance. Similarly, $\bar{s}$ is the prior mean of the speed associated with its prior variance $\sigma_{s}^{2}$, and $\sigma_{c}^{2}$ is the prior course variance. The predicted estimates ${\hat{e}}_{k|k-1}^{\left(i\right)}$ and $J_{k|k-1}^{\left(i\right)}$ are obtained via the Kalman prediction:

(19) $$\begin{array}{c} {\hat{e}}_{k|k-1}^{\left(i\right)}=\Phi {\hat{e}}_{k-1}^{\left(i\right)}, \end{array}$$

(20) $$\begin{array}{c} J_{k|k-1}^{\left(i\right)}=\Phi J_{k|k-1}^{\left(i\right)}\Phi^{\prime }+Q_{k-1}. \end{array}$$

The target state is augmented by a binary variable *β*, so we may distinguish between the surviving components and the birth components:

(21) $$\begin{array}{c} v_{k|k-1}\left(e,\beta \right)=\left\{\begin{array}{c} \sum_{i=1}^{J_{k-1}}\omega_{k|k-1}^{\left(i\right)}N\left(e;{\hat{e}}_{k|k-1}^{\left(i\right)},J_{k|k-1}^{\left(i\right)}\right),\beta =0\\ \Psi \left(e;\gamma_{k}\left(\theta ,r,c,s\right)\right),\beta =1 \end{array}\right. \end{array}$$

where $\omega_{k|k-1}^{\left(i\right)}=P_{S,k}\omega_{k-1}^{\left(i\right)}$. As noted in [34], the surviving and birth components are separated to avoid biasing the cardinality estimates.

### 3.2. GMM Likelihood Approximation

In GM-PHD filters, the likelihood function $g_{k}\left(w|e\right)$ is always modeled as a single Gaussian distribution. However, it is approximated by a Gaussian mixture in the proposed GMM-PHD filter.

Though the measurement noise is assumed to be Gaussian, the measurement uncertainty is non-Gaussian (non-ellipse) in Cartesian coordinates. The GMM measurement presentation first divides the non-elliptical measurement uncertainty into several segments. Then, each segment is modeled by one Gaussian distribution, and the whole measurement uncertainty can be approximated by a Gaussian mixture.

Suppose the measurement uncertainty in Cartesian coordinates is determined by the range interval $\left[r_{k,\min},r_{k,\max}\right]$ and the measurement $\theta_{k}^{t}$ with standard deviation $\sigma_{\theta }$. The range interval is divided into $A_{k}$ subintervals, given by [35,36]:

(22) $$\frac{r_{k,a+1}}{r_{k,a}}=\tau_{k};\;a=1,\ldots ,A_{k},$$

where:

(23) τk=rk,maxrk,min11BkAk.

Then, each segment *a* can be determined by $r_{k,a},r_{k,a+1},\theta_{k}^{t}-\sigma_{\theta },\theta_{k}^{t}+\sigma_{\theta }$ in polar coordinates. Let $\bar{r}=\left(r_{k,a}+r_{k,a+1}\right)/2$ and $\Delta r=\left(r_{k,a+1}-r_{k,a}\right)/2$, then the segment *a* is approximated by a Gaussian distribution with mean ${\hat{w}}_{k,a}$ and covariance $R_{k,a}$ in Cartesian coordinates:

(24) $${\hat{w}}_{k,a}=\left[\begin{array}{c} x_{k}^{s}\\ y_{k}^{s} \end{array}\right]+\bar{r}\left[\begin{array}{c} \sin\left(\theta_{k}^{t}\right)\\ \cos\left(\theta_{k}^{t}\right) \end{array}\right],$$

(25) $$R_{k,a}=\phi \left[\begin{array}{cc} {\left(\Delta r\right)}^{2} & 0\\ 0 & {\bar{r}}^{2}\sigma_{\theta }^{2} \end{array}\right]\phi^{\prime },$$

where:

(26) $$\phi =\left[\begin{array}{cc} \sin\left(\theta_{k}^{t}\right) & -\cos\left(\theta_{k}^{t}\right)\\ \cos\left(\theta_{k}^{t}\right) & \sin\left(\theta_{k}^{t}\right) \end{array}\right].$$

Obviously, the area of each segment is different, and the weight of the segment *a* is proportional to its area, given by [35]:

(27) $$\lambda_{k,a}=\frac{\sqrt{\det\left(R_{k,a}\right)}}{\sum_{a=1}^{A_{k}}\sqrt{\det\left(R_{k,a}\right)}},$$

and

(28) $$\sum_{a=1}^{A_{k}}\lambda_{k,a}=1.$$

Then, the measurement likelihood function in Cartesian coordinate $\left(\beta =0\right)$ is approximated as a Gaussian mixture:

(29) $$\begin{array}{c} g_{k}\left(w|e,\beta =0\right)\approx C_{k}\sum_{a=1}^{A_{k}}\lambda_{k,a}N\;\left({\hat{w}}_{k,a};He_{k},R_{k,a}\right), \end{array}$$

where, $H=\left[\begin{array}{cccc} 1 & 0 & 0 & 0\\ 0 & 1 & 0 & 0 \end{array}\right]$ is the observation matrix and the constant $C_{k}$ is calculated as [37]:

(30) $$C_{k}=\int_{r_{k,\min}}^{r_{k,\max}}rdr=\frac{r_{k,\max}^{2}-r_{k,\min}^{2}}{2}.$$

Since the measurement noise is modeled as Gaussian, the likelihood function in polar coordinates $\left(\beta =1\right)$ is expressed as:

(31) $$\begin{array}{c} g_{k}\left(w|e,\beta =1\right)=N\;\left(\theta_{k}^{t};\theta ,\sigma_{\theta }^{2}\right) \end{array}$$

**Figure 1:**
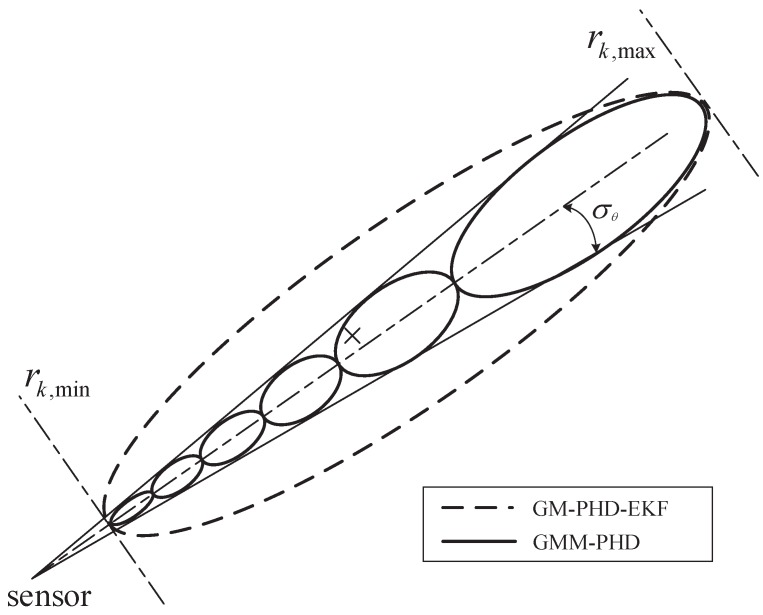
An example of GMM presentation.

Figure 1 gives an example of the GMM presentation. In the figure, the measurement uncertainty of the GMM-PHD is approximated by six measurement components (shown as the solid ellipses), and that of the GM-PHD-EKF is modeled by a single Gaussian distribution (shown as the dashed ellipse). Actually, the measurement uncertainty of the GM-PHD-UKF is also approximated by one Gaussian distribution. The true target is displayed by a cross. It is obvious that the measurement likelihood function approximated using Gaussian mixtures in the GMM-PHD is much more accurate than the approximation with one Gaussian in the GM-PHD-EKF. Thus, the GMM-PHD can perform with a much higher tracking accuracy than the GM-PHD-EKF and the GM-PHD-UKF.

### 3.3. Intensity Update

New targets are assumed to be always detected at their time of birth. Furthermore, we also assume that the detection probability for a surviving target is independent of their state and:

(32) $$\begin{array}{c} P_{D,k}\left(e,\beta \right)=\left\{\begin{array}{c} P_{D,k},\beta =0\\ 1,\beta =1 \end{array}\right.. \end{array}$$

According to Equation (14), the posterior intensity at time *k* is given by:

(33) $$\begin{array}{c} v_{k|k}\left(e,\beta =0\right)\\ =\left[1-P_{D,k}\right]\sum_{i=1}^{J_{k-1}}\omega_{k|k-1}^{\left(i\right)}N\left(e;{\hat{e}}_{k|k-1}^{\left(i\right)},J_{k|k-1}^{\left(i\right)}\right)\\ \;+\sum_{w\in W_{k}}\sum_{i=1}^{J_{k-1}}\frac{\begin{array}{c} P_{D,k}C_{k}\sum_{a=1}^{A_{k}}\lambda_{k,a}N\;\left({\hat{w}}_{k,a};He_{k},R_{k,a}\right)\omega_{k|k-1}^{\left(i\right)}N\left(e;{\hat{e}}_{k|k-1}^{\left(i\right)},J_{k|k-1}^{\left(i\right)}\right) \end{array}}{\begin{array}{c} \kappa_{k}\left(w\right)+\int P_{D,k}\left(e\right)g_{k}\left(w|e\right)v_{k|k-1}\left(e\right)de \end{array}}\\ \;\;=\left[1-P_{D,k}\right]\sum_{i=1}^{J_{k-1}}\omega_{k|k-1}^{\left(i\right)}N\left(e;{\hat{e}}_{k|k-1}^{\left(i\right)},J_{k|k-1}^{\left(i\right)}\right)\\ \;+\sum_{w\in W_{k}}\sum_{i=1}^{J_{k-1}}\sum_{a=1}^{A_{k}}\frac{\begin{array}{c} P_{D,k}C_{k}\lambda_{k,a}N\left({\hat{w}}_{k,a};He_{k},R_{k,a}\right)\omega_{k|k-1}^{\left(i\right)}N\left(e;e_{k|k-1}^{\left(i\right)},J_{k|k-1}^{\left(i\right)}\;\right) \end{array}}{\begin{array}{c} \kappa_{k}\left(w\right)+\int P_{D,k}\left(e\right)g_{k}\left(w|e\right)\cdot v_{k|k-1}\left(e\right)de \end{array}}, \end{array}$$

and:

(34) $$\begin{array}{c} v_{k|k}\left(e,\beta =1\right)\\ =\sum_{w\in W_{k}}\frac{\begin{array}{c} g_{k}\left(w|e\right)\Psi \left(e;\gamma_{k}\left(\theta ,r,c,s\right)\right) \end{array}}{\begin{array}{c} \kappa_{k}\left(w\right)+\int P_{D,k}\left(e\right)g_{k}\left(w|e\right)v_{k|k-1}\left(e\right)de \end{array}}\\ =\sum_{w\in W_{k}}\frac{\begin{array}{c} \Psi \left(e;g_{k}\left(w|e\right)\gamma_{k}\left(\theta ,r,c,s\right)\right) \end{array}}{\begin{array}{c} \kappa_{k}\left(w\right)+\int P_{D,k}\left(e\right)g_{k}\left(w|e\right)v_{k|k-1}\left(e\right)de \end{array}}. \end{array}$$

In the first step of Equation (34), the likelihood function $g_{k}\left(w|e\right)$ is expressed by a Gaussian distribution Equation (31), but not a Gaussian mixture Equation (29), as the target birth model $\gamma_{k}\left(\theta ,r,c,s\right)$ is given in polar coordinates (uniform across the bearing space and Gaussian in the range and velocity). As both of them are expressed in the same polar coordinates, we have:

(35) $$g_{k}\left(w|e\right)\Psi \left(e;\gamma_{k}\left(\theta ,r,c,s\right)\right)=\Psi \left(e;g_{k}\left(w|e\right)\gamma_{k}\left(\theta ,r,c,s\right)\right).$$

In the above equation,

(36) $$\begin{array}{c} \varphi_{k}\left(\theta ,r,c,s\right)≜g_{k}\left(w|e\right)\gamma_{k}\left(\theta ,r,c,s\right)\\ =N\;\left(\theta_{k}^{t};\theta ,\sigma_{\theta }^{2}\right)\omega_{k}^{b}U\;\left(\theta ;H_{\theta }\right)N\left(r;\bar{r},\sigma_{r}^{2}\right)N\left(c;\theta -\pi ,\sigma_{c}^{2}\right)\\ \;\cdot N\left(s;\bar{s},\sigma_{s}^{2}\right)\\ =\omega_{k}^{b}\frac{1_{H}\left(\theta \right)}{V_{H}}N\;\left(\theta ;\theta_{k}^{t},\sigma_{\theta }^{2}\right)N\left(r;\bar{r},\sigma_{r}^{2}\right)N\left(c;\theta -\pi ,\sigma_{c}^{2}\right)\\ \;\cdot N\left(s;\bar{s},\sigma_{s}^{2}\right)\\ \approx \frac{\omega_{k}^{b}}{2\pi }N\;\left(\theta ;\theta_{k}^{t},\sigma_{\theta }^{2}\right)N\left(r;\bar{r},\sigma_{r}^{2}\right)N\left(c;\theta -\pi ,\sigma_{c}^{2}\right)N\left(s;\bar{s},\sigma_{s}^{2}\right), \end{array}$$

where $1_{H}\left(\theta \right)$ is the indicator function of the bearing space region $H_{\theta }$ and $V_{H}$ is the volume of $H_{\theta }$, and we use the approximation:

(37) $$1_{H}\left(\theta \right)N\;\left(\theta ;\theta_{k}^{t},\sigma_{\theta }^{2}\right)\approx N\;\left(\theta ;\theta_{k}^{t},\sigma_{\theta }^{2}\right)$$

which is reasonable in practice by assuming that $\sigma_{\theta }$ is small compared to the region $H_{\theta }$. The transformation function $\Psi \left(e;\varphi_{k}\left(\theta ,r,c,s\right)\right)$ transforms the function $\varphi_{k}\left(\theta ,r,c,s\right)$ from polar coordinates to Cartesian coordinates, given by:

(38) $$\Psi \left(e;\varphi_{k}\left(\theta ,r,c,s\right)\right)\approx \frac{\omega_{k}^{b}}{2\pi }\sum_{a=1}^{A_{k}}\lambda_{k,a}N\left(e;{\tilde{e}}_{k}\left(\theta_{k}^{t}\right),{\tilde{J}}_{k}\left(\theta_{k}^{t}\right)\right),$$

where the weight $\lambda_{k,a}$ is given by Equation (27), the position part of the mean ${\tilde{e}}_{k}\left(\theta_{k}^{t}\right)$ and the covariance ${\tilde{J}}_{k}\left(\theta_{k}^{t}\right)$ are given by Equations (24) and (25), respectively, and the velocity part is calculated by the approximation [38,39]:

(39) $${\tilde{e}}_{k}\left(\theta_{k}^{t}\right)=\left[\begin{array}{c} x_{k}^{s}+\bar{r}\sin\left(\theta_{k}^{t}\right)\\ y_{k}^{s}+\bar{r}\cos\left(\theta_{k}^{t}\right)\\ \bar{s}\sin\left(\theta_{k}^{t}-\pi \right)\\ \bar{s}\cos\left(\theta_{k}^{t}-\pi \right) \end{array}\right],$$

(40) $${\tilde{J}}_{k}\left(\theta_{k}^{t}\right)=\left[\begin{array}{cccc} P_{xx} & P_{xy} & 0 & 0\\ P_{yx} & P_{yy} & 0 & 0\\ 0 & 0 & P_{\dot{x}\dot{x}} & P_{\dot{x}\dot{y}}\\ 0 & 0 & P_{\dot{y}\dot{x}} & P_{\dot{y}\dot{y}} \end{array}\right],$$

where:

(41) $$P_{xx}={\left(\Delta r\right)}^{2}\;\sin^{2}\;\left(\theta_{k}^{t}\;\right)+{\bar{r}}^{2}\sigma_{\theta }^{2}\cos^{2}\;\left(\theta_{k}^{t}\right),$$

(42) $$P_{yy}={\left(\Delta r\right)}^{2}\;\cos^{2}\;\left(\theta_{k}^{t}\;\right)+{\bar{r}}^{2}\sigma_{\theta }^{2}\sin^{2}\;\left(\theta_{k}^{t}\right),$$

(43) $$P_{xy}=P_{yx}=\frac{1}{2}\sin2\theta_{k}^{t}\left[{\left(\Delta r\right)}^{2}-{\bar{r}}^{2}\sigma_{\theta }^{2}\right],$$

(44) $$P_{\dot{x}\dot{x}}=\sigma_{s}^{2}\sin^{2}\left(\theta_{k}^{t}-\pi \right)+\sigma_{c}^{2}{\bar{s}}^{2}\cos^{2}\left(\theta_{k}^{t}-\pi \right),$$

(45) $$P_{\dot{x}\dot{x}}=\sigma_{s}^{2}\cos^{2}\left(\theta_{k}^{t}-\pi \right)+\sigma_{c}^{2}{\bar{s}}^{2}\sin^{2}\left(\theta_{k}^{t}-\pi \right),$$

(46) $$P_{\dot{x}\dot{y}}=P_{\dot{y}\dot{x}}=\frac{1}{2}\sin\left(2\left(\theta_{k}^{t}-\pi \right)\right)\left[\sigma_{s}^{2}-\sigma_{c}^{2}{\bar{s}}^{2}\right].$$

In the denominator of Equations (33) and (34), we have the following:

(47) $$\begin{array}{c} \int P_{D,k}\left(e\right)g_{k}\left(w|e\right)v_{k|k-1}\left(e\right)de\\ =\;\int \int \;P_{D,k}\left(e,\beta \right)g_{k}\left(w|e,\beta \right)v_{k|k-1}\left(e,\beta \right)ded\beta \\ =\int \sum_{\beta =0}^{1}P_{D,k}\left(e,\beta \right)g_{k}\left(w|e,\beta \right)v_{k|k-1}\left(e,\beta \right)de\\ =\int P_{D,k}C_{k}\sum_{a=1}^{A_{k}}\lambda_{k,a}N\left({\hat{w}}_{k,a};He_{k},R_{k,a}\right)\sum_{i=1}^{J_{k-1}}\omega_{k|k-1}^{\left(i\right)}N\left(e;{\hat{e}}_{k|k-1}^{\left(i\right)},J_{k|k-1}^{\left(i\right)}\right)de\\ +\int \Psi \left(e;g_{k}\left(w|e\right)\gamma_{k}\left(\theta ,r,c,s\right)\right)de\\ =P_{D,k}C_{k}\sum_{a=1}^{A_{k}}\sum_{i=1}^{J_{k-1}}\lambda_{k,a}\omega_{k|k-1}^{\left(i\right)}N\left({\hat{w}}_{k,a};H{\hat{e}}_{k|k-1}^{\left(i\right)},S_{k|k-1}^{\left(i\right)}\right)\int N\left(e;{\hat{e}}_{k|k}^{\left(i,a\right)},J_{k|k}^{\left(i,a\right)}\right)de\\ +\int \frac{\omega_{k}^{b}}{2\pi }\sum_{a=1}^{A_{k}}\lambda_{k,a}N\left(e;{\tilde{e}}_{k}\left(\theta_{k}^{t}\right),{\tilde{J}}_{k}\left(\theta_{k}^{t}\right)\right)de\\ =P_{D,k}C_{k}\sum_{a=1}^{A_{k}}\sum_{i=1}^{J_{k-1}}\lambda_{k,a}\omega_{k|k-1}^{\left(i\right)}q_{k}^{\left(i\right)}\left({\hat{w}}_{k,a}\right)\;+\;\frac{\omega_{k}^{b}}{2\pi }, \end{array}$$

where $q_{k}^{\left(i\right)}\left({\hat{w}}_{k,a}\right)=N\left({\hat{w}}_{k,a};H{\hat{e}}_{k|k-1}^{\left(i\right)},S_{k|k-1}^{\left(i\right)}\right)$ is the likelihood of measurement component ${\hat{w}}_{k,a}$ against the component *i* with the innovation covariance:

(48) $$S_{k|k-1}^{\left(i\right)}=HJ_{k|k-1}^{\left(i\right)}H^{\prime }+R_{k,a},$$

and the updated target states and corresponding covariance are given by:

(49) $${\hat{e}}_{k|k}^{\left(i,a\right)}={\hat{e}}_{k|k-1}^{\left(i\right)}+K_{k}^{\left(i\right)}\left({\hat{w}}_{k,a}-H{\hat{e}}_{k|k-1}^{\left(i\right)}\right),$$

(50) $$J_{k|k}^{\left(i,a\right)}=J_{k|k-1}^{\left(i\right)}-K_{k}^{\left(i\right)}HJ_{k|k-1}^{\left(i\right)},$$

with the Kalman gain:

(51) $$K_{k}^{\left(i\right)}=J_{k|k-1}^{\left(i\right)}H^{\prime }{\left(S_{k|k-1}^{\left(i\right)}\right)}^{-1}.$$

The following approximation for the posterior intensity can be obtained:

(52) $$\begin{array}{c} v_{k|k}\left(e,\beta =0\right)\;\\ \;=\left[1-P_{D,k}\right]\sum_{i=1}^{J_{k-1}}\omega_{k|k-1}^{\left(i\right)}N\left(e;{\hat{e}}_{k|k-1}^{\left(i\right)},J_{k|k-1}^{\left(i\right)}\right)\\ \;+\sum_{w\in W_{k}}\sum_{i=1}^{J_{k-1}}\sum_{a=1}^{A_{k}}\frac{\begin{array}{c} P_{D,k}C_{k}\lambda_{k,a}N\left({\hat{w}}_{k,a};He_{k},R_{k,a}\right)\omega_{k|k-1}^{\left(i\right)}N\left(e;{\hat{e}}_{k|k-1}^{\left(i\right)},J_{k|k-1}^{\left(i\right)}\right) \end{array}}{\begin{array}{c} \kappa_{k}\left(w\right)+P_{D,k}C_{k}\sum_{a=1}^{A_{k}}\sum_{i=1}^{J_{k-1}}\lambda_{k,a}\omega_{k|k-1}^{\left(i\right)}q_{k}^{\left(i\right)}\left({\hat{w}}_{k,a}\right)\;+\;\frac{\omega_{k}^{b}}{2\pi } \end{array}}\\ \;=\sum_{i=1}^{J_{k-1}}\omega_{m,k}^{\left(i\right)}N\left(e;{\hat{e}}_{k|k-1}^{\left(i\right)},J_{k|k-1}^{\left(i\right)}\right)\;+\sum_{w\in W_{k}}\sum_{i=1}^{J_{k-1}}\sum_{a=1}^{A_{k}}\omega_{s,k}^{\left(i,a\right)}N\left(e;{\hat{e}}_{k|k}^{\left(i,a\right)},J_{k|k}^{\left(i,a\right)}\right), \end{array}$$

and:

(53) $$\begin{array}{c} v_{k|k}\left(e,\beta =1\right)\\ \;\;=\sum_{w\in W_{k}}\sum_{a=1}^{A_{k}}\frac{\frac{\omega_{k}^{b}}{2\pi }\lambda_{k,a}N\left(e;{\tilde{e}}_{k}\left(\theta_{k}^{t}\right),{\tilde{J}}_{k}\left(\theta_{k}^{t}\right)\right)}{\begin{array}{c} \kappa_{k}\left(w\right)+P_{D,k}C_{k}\sum_{a=1}^{A_{k}}\sum_{i=1}^{J_{k-1}}\lambda_{k,a}\omega_{k|k-1}^{\left(i\right)}q_{k}^{\left(i\right)}\left({\hat{w}}_{k,a}\right)\;+\;\frac{\omega_{k}^{b}}{2\pi } \end{array}}\\ \;=\sum_{w\in W_{k}}\sum_{a=1}^{A_{k}}\omega_{b,k}N\left(e;{\tilde{e}}_{k}\left(\theta_{k}^{t}\right),{\tilde{J}}_{k}\left(\theta_{k}^{t}\right)\right), \end{array}$$

where:

(54) $$\omega_{m,k}^{\left(i\right)}=\left[1-P_{D,k}\right]\omega_{k|k-1}^{\left(i\right)},$$

(55) $$\omega_{s,k}^{\left(i,a\right)}=\frac{P_{D,k}C_{k}\lambda_{k,a}\omega_{k|k-1}^{\left(i\right)}q_{k}^{\left(i\right)}\left({\hat{w}}_{k,a}\right)}{\begin{array}{c} \kappa_{k}\left(w\right)+P_{D,k}C_{k}\sum_{a=1}^{A_{k}}\sum_{i=1}^{J_{k-1}}\lambda_{k,a}\omega_{k|k-1}^{\left(i\right)}q_{k}^{\left(i\right)}\left({\hat{w}}_{k,a}\right)\;+\;\frac{\omega_{k}^{b}}{2\pi } \end{array}},$$

(56) ωb,ka=λk,aωkbλk,aωkbVH2πκkw+PD,kCk∑a=1Ak∑i=1Jk-1λk,aωk|k-1iqkiw^k,a+ωkb2π.

### 3.4. Component Management and State Extraction

Just like the GM-PHD filter, the number of Gaussian components of the updated intensity exponentially increases in time. To reduce the computational load, same techniques (component merging and pruning) in [27,31] are also implemented in the GMM-PHD filter. If the weight of a Gaussian component is lower than the preset threshold, it will be discarded. When some of components are close enough, they will be merged into one Gaussian component. If the total number of Gaussian components is bigger than the maximum value $M_{k}$, only $M_{k}$ components with high probability will be retained. More details about component management can be found in [31]. The state extraction is also the same as the method in [31]. If the weights are bigger than a threshold, the corresponding states are extracted as the outputs of the filter.

## 4. Simulation Experiments

To illustrate the improvements of the proposed GMM-PHD filter, we compare it with the GM-PHD-EKF and the GM-PHD-UKF, which also use a partially-uniform birth model. The GM-PHD-EKF and GM-PHD-UKF were introduced in [8] for a challenging bearings-only multi-target scenario.

### 4.1. Simulation Scenarios

#### 4.1.1. Case 1

**Figure 2:**
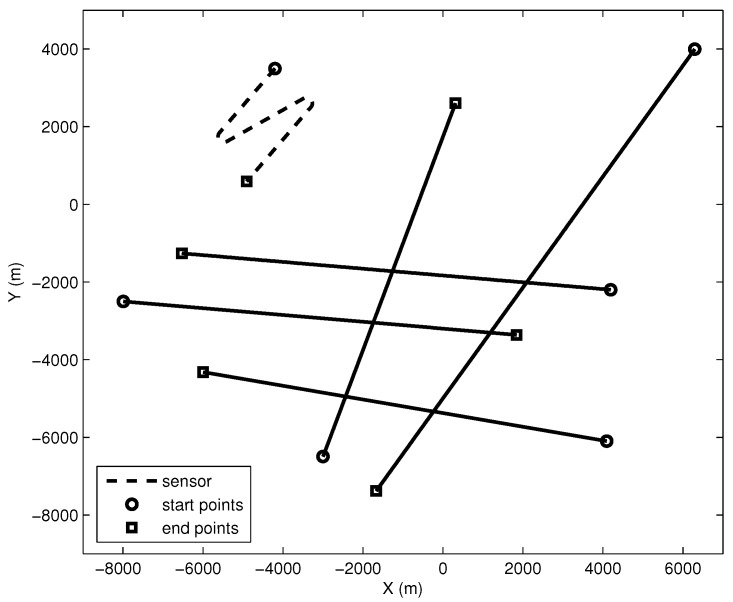
The geometry of sensor and targets.

The sensor is located on a maneuvering platform with the initial position $\left[-4200\;m,3500\;m\right]$. The sensor platform moves at a speed of five knots and changes course two times to ensure the observability of the bearings-only target tracking. Note that the sensor should change course again to track the targets that appear after the second sensor maneuvering. The initial course of the sensor is $220^{\circ }$and changes to $60^{\circ }$ from 14 min to 17 min. The second course change occurs from 31 min to 34 min, with the course changed from $60^{\circ }$ to $220^{\circ }$. Note that the clockwise rotation from the positive Y-axis is considered to be positive. A time-varying number of targets is contained in the scenario, and the motion profile of targets is presented in Table 1. The starting positions of Targets #1and#2 are $\left[-8000\;m,-2500\;m\right]$ and $\left[-3000\;m,-6500\;m\right]$, respectively. Similarly, the initial positions of Targets #3and#4 are $\left[4100\;m,-6100\;m\right]$ and $\left[4200\;m,-2200\;m\right]$, respectively. Finally, the travel of Target #5 starts at $\left[6300\;m,4000\;m\right]$. An illustration of the sensor and the targets without process noise and clutter is presented in Figure 2.

In the simulation, the sampling time of the sensor is 10 s, and the total simulation time is 3000 s. The sensor generates measurements with a standard deviation $\sigma_{\theta }=1^{\circ }$, and the survival probability of all targets is also the same, $P_{S,k}=0.98$. The number of clutter measurements is assumed to follow a Poisson distribution with a mean of 15. The detection probability in Case 1 is 0.95. The clutter measurements are distributed uniformly across the whole bearing space $\left[0,\;2\pi \right]$. The PSD is $q=2.5\times 10^{-5}\;m^{2}/s^{4}$.

In the target birth model, the prior selected parameters are the same for all filters. The prior target course is $\theta_{k}^{t}-\pi$ with standard deviation $\sigma_{c}=50^{\circ }$. The prior target speed is $\bar{s}=10$ knots with the standard deviation $\sigma_{s}=4$ knots. The prior target range for the GM-PHD-EKF and the GM-PHD-UKF is $\bar{r}=12,000$ m with standard deviation $\sigma_{r}=4000$ m. The range interval for the GMM-PHD is $\left[300\;m,\;18000\;m\right]$. The number of measurement components in each scan is $A_{k}=8$. The birth intensity is set to $\omega_{k}^{b}=0.05$ for all algorithms. The thresholds for the component merging and pruning are four and $10^{-5}$, respectively. Further, all filters have the maximum number of the Gaussian components, $M_{k}=100$.

#### 4.1.2. Case 2

**Figure 3:**
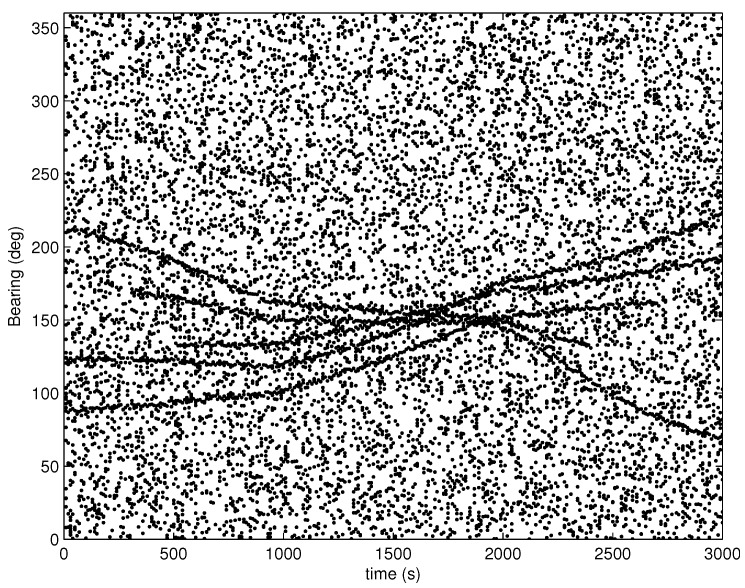
An example of measurements.

In this case, a more challenging scenario with higher clutter intensity and lower detection probability is considered. Compared with Case 1, the mean number of clutter measurements is increased to 30, and the detection probability is decreased to 0.85. The other parameters of Case 2 are the same as those of Case 1. An example of the measurements of Case 2 is shown in Figure 3.

#### 4.1.3. Case 3

In this case, some selected parameters of filters are changed to show whether the proposed GMM-PHD also has better performance than the GM-PHD-EKF and the GM-PHD-UKF. The survival probability $P_{S,k}$ in Case 3 is changed to 0.95. The birth intensity $\omega_{k}^{b}$ is set as 0.01 in all filters. Besides, the maximum number of the Gaussian components $M_{k}$ is increased to 150 in each filter. The other parameters of Case 3 are also the same as those of Case 1.

#### 4.1.4. Case 4

**Figure 4:**
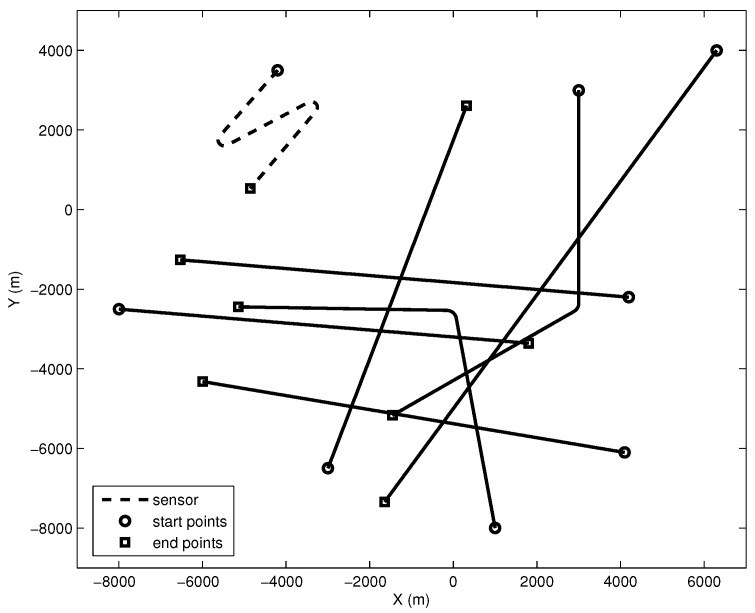
The geometry of the sensor and targets.

In this case, two maneuvering Targets #6and#7 are added to Case 1. The starting positions of Targets #6and#7 are $\left[1000\;m,-8000\;m\right]$ and $\left[3000\;m,3000\;m\right]$, respectively. The course of Target #6 is changed from $350^{\circ }$ to $270^{\circ }$ at 25 min. The initial course of Target #7 is $180^{\circ }$ and changes to $240^{\circ }$ at 28 min. The survival times of Targets #6and#7 are $\left[200\;s,\;2800\;s\right]$ and $\left[400\;s,\;3000\;s\right]$, respectively. The speed of two targets is eight knots. The other parameters of Case 4 are also the same as those of Case 1. An illustration of the sensor and the targets without process noise and clutter in Case 4 is presented in Figure 4.

### 4.2. Simulation Results and Analyses

**Figure 5:**
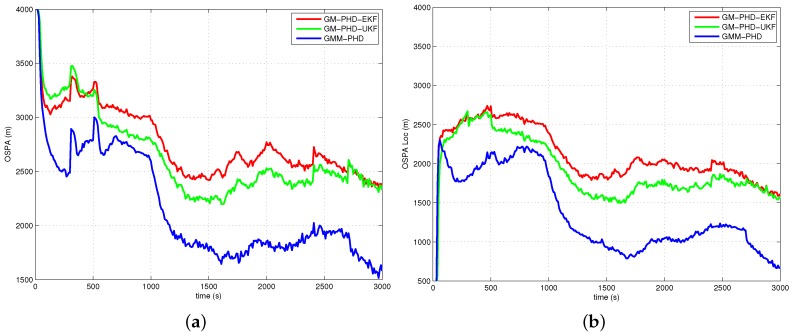

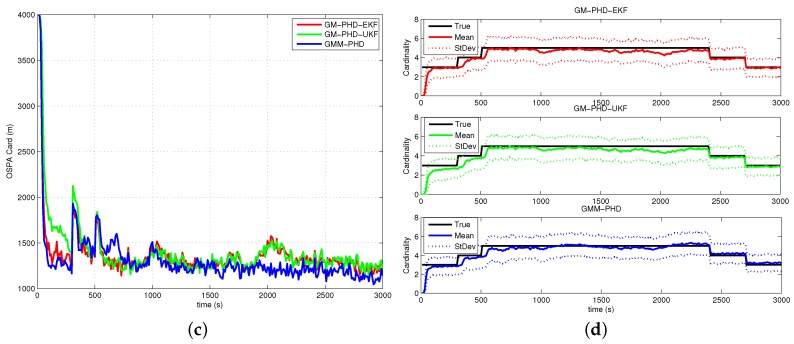
Results of Case 1: (**a**) Optimal subpattern assignment (OSPA) distance; (**b**) OSPA localization; (**c**) OSPA cardinality; (**d**) Cardinality statistics.

**Figure 6:**
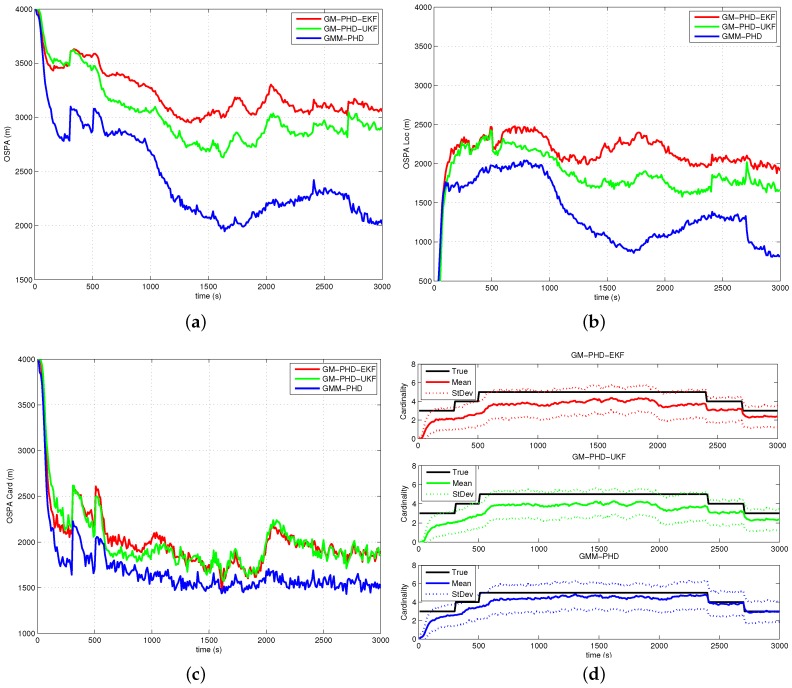
Results of Case 2: (**a**) OSPA distance; (**b**) OSPA localization; (**c**) OSPA cardinality; (**d**) Cardinality statistics.

**Figure 7:**
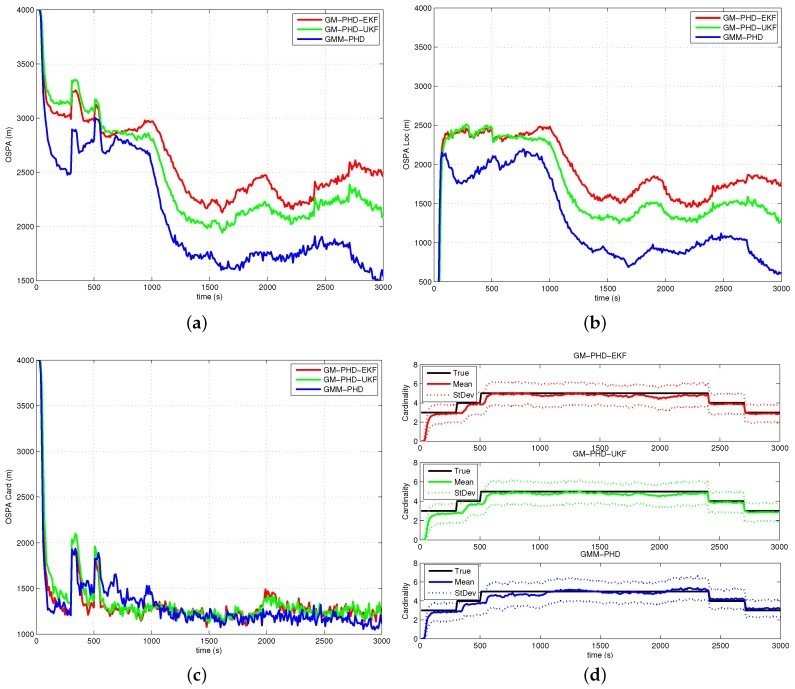
Results of Case 3: (**a**) OSPA distance; (**b**) OSPA localization; (**c**) OSPA cardinality; (**d**) Cardinality statistics.

**Figure 8:**
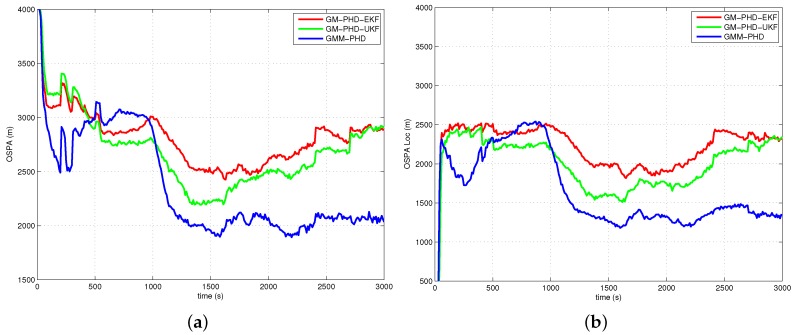

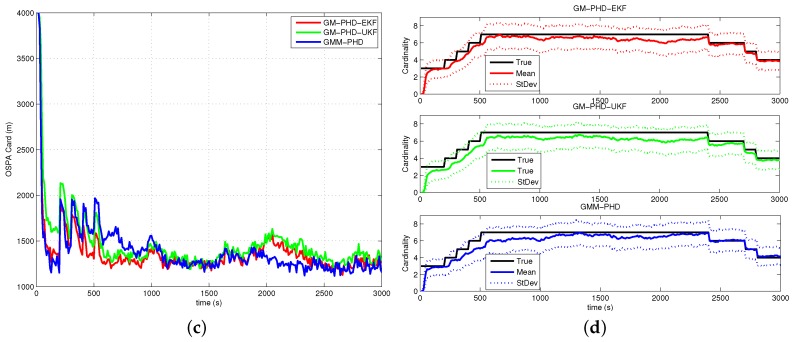
Results of Case 4: (**a**) OSPA distance; (**b**) OSPA localization; (**c**) OSPA cardinality; (**d**) Cardinality statistics.

**Figure 9:**
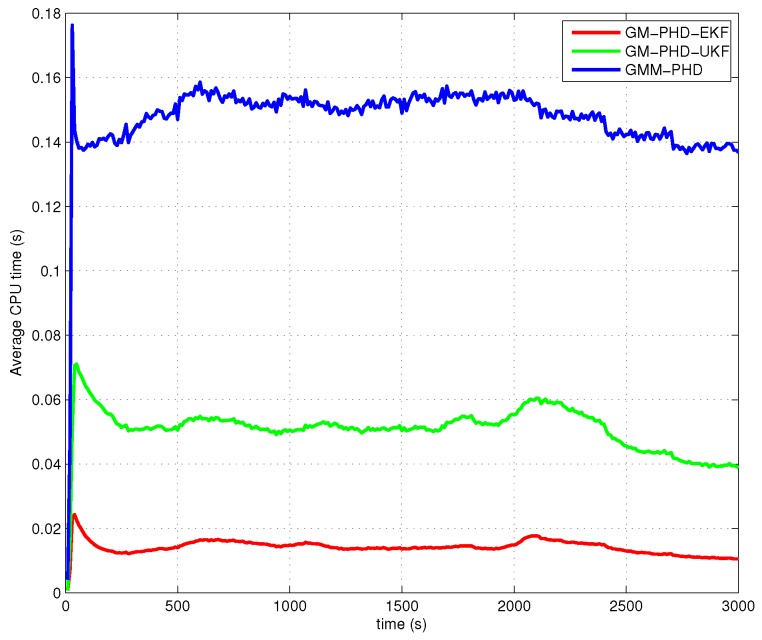
Execution time.

The performances of all filters are compared with the average OSPA metric (with order two and cutoff 4000 m) over 500 Monte Carlo runs. The simulation results of Case 1, Case 2, Case 3 and Case 4 are presented in Figure 5, Figure 6, Figure 7 and Figure 8, respectively. The OSPA distance consists of two components: localization and cardinality. Figure 5a, Figure 6a, Figure 7a and Figure 8a show OSPA distances of three filters. The OSPA localizations are presented in Figure 5b, Figure 6b, Figure 7b and Figure 8b. The OSPA cardinalities are shown in Figure 5c, Figure 6c, Figure 7c and Figure 8c. The cardinality statistics are also considered and are shown in Figure 5d, Figure 6d, Figure 7d and Figure 8d. The execution time of different filters for Case 2 is also compared and shown in Figure 9.

In Figure 5a, Figure 6a, Figure 7a and Figure 8a, the OSPA distance of the GM-PHD-UKF is slightly lower than that of the GM-PHD-EKF. The GMM-PHD performs significantly better than the other two filters. Before 1000 s, the target states are unobservable, and all of the filters have a large OSPA distance error. After the sensor maneuver, the target states become observable, and the OSPA distance decreases sharply. From 1500 s to 2500 s, the OSPA distances of all filters show a little bit of an increase as the measurements overlap, which makes it hard for the filters to estimate the target states.

In Figure 5b, Figure 6b, Figure 7b and Figure 8b, the OSPA localization of the GMM-PHD is much lower than those of the GM-PHD-EKF and the GM-PHD-UKF after the target states become observable. Especially in Case 4, which contains two maneuver targets, the OSPA localization errors of the GM-PHD-EKF and the GM-PHD-UKF have significant increases after target maneuvering, as shown in Figure 8b. However, the proposed GMM-PHD can keep a high tracking accuracy as the likelihood function is modeled by Gaussian mixtures, which can quickly responds to the target course change. The performance of the GM-PHD-UKF is also better than the GM-PHD-EKF on the OSPA localization metric in all cases.

In Case 1, Case 3 and Case 4, the three filters have almost the same OSPA cardinality error as shown in Figure 5c, Figure 7c and Figure 8c. Additionally, the three filters have accurate cardinality estimates in Figure 5d, Figure 7d and Figure 8d. For Case 2, the GMM-PHD has some improvement of the OSPA cardinality criteria compared to the GM-PHD-EKF and the GM-PHD-UKF in Figure 6c. Furthermore, the cardinality statistic of the GMM-PHD is more accurate than those of other two filters in Figure 6d. From Figure 5d, Figure 6d, Figure 7d and Figure 8d, three filters suffer from a delay in detecting a new target as the targets are not observable before the sensor maneuver.

According to the discussion above, the improvement of the GMM-PHD is mainly reflected in increasing the tracking accuracy of the target states, but not the cardinality estimation of the targets, for Case 1, Case 3 and Case 4. When the simulation situation becomes worse (the clutter intensity becomes bigger, and the probability of target detection decreases) in Case 2, the GMM-PHD shows improvement on both the track accuracy and the cardinality estimate. As the measurement likelihood is approximated by a Gaussian mixture, the number of track components in the GMM-PHD filter is bigger than for the GM-PHD-EKF and the GM-PHD-UKF. The execution time of the GMM-PHD filter is slightly larger than the other two filters, as shown in Figure 9. However, the execution times of these filters are much smaller than the real time. Note that the computational load of each filter becomes heavier if the number of targets increases, as the maximum number $M_{k}$ should be set bigger to ensure good performances.

## 5. Conclusions

For bearings-only multi-target tracking with clutter and missed detections, the PHD filter is popular and known to be effective. As there is no closed form solution to the original PHD recursion, the Gaussian mixture assumption is applied, and the GM-PHD filter was designed. However, the likelihood function of the GM-PHD filter is always modeled by a Gaussian distribution. In this paper, the GMM-PHD filter was proposed in a way that the likelihood function, as well as the posterior intensity are modeled by Gaussian mixtures. In this way, the likelihood function of the GMM-PHD can be approximated more accurately than those of the GM-PHD-EKF and the GM-PHD-UKF. The simulation results show that the proposed algorithm has significant improvement of target state estimation for challenging bearings-only multi-target tracking scenarios at the expense of computational resources. Besides, the targets appear at several fixed points in the standard form of the GM-PHD filter. The GMM-PHD was derived by using a partially uniform target birth model that can reduce the number of parameters that must be chosen by the end user.

## Figures and Tables

**Table 1:** Motion profile of targets.

Target	Survival Time (s)	Course (degree)	Speed (knots)
#1	$\left[0,\;2400\right]$	95	8
#2	$\left[300,\;3000\right]$	20	7
#3	$\left[500,\;3000\right]$	280	8
#4	$\left[0,\;3000\right]$	275	7
#5	$\left[0,\;2700\right]$	215	10
